# Pupillary Responses to Robotic and Human Emotions: The Uncanny Valley and Media Equation Confirmed

**DOI:** 10.3389/fpsyg.2018.00774

**Published:** 2018-05-23

**Authors:** Anne Reuten, Maureen van Dam, Marnix Naber

**Affiliations:** Experimental Psychology, Helmholtz Institute, Faculty of Social Sciences, Utrecht University, Utrecht, Netherlands

**Keywords:** robots, emotion, pupil, facial expressions, social acceptance

## Abstract

Physiological responses during human–robots interaction are useful alternatives to subjective measures of uncanny feelings for nearly humanlike robots (uncanny valley) and comparable emotional responses between humans and robots (media equation). However, no studies have employed the easily accessible measure of pupillometry to confirm the uncanny valley and media equation hypotheses, evidence in favor of the existence of these hypotheses in interaction with emotional robots is scarce, and previous studies have not controlled for low level image statistics across robot appearances. We therefore recorded pupil size of 40 participants that viewed and rated pictures of robotic and human faces that expressed a variety of basic emotions. The robotic faces varied along the dimension of human likeness from cartoonish to humanlike. We strictly controlled for confounding factors by removing backgrounds, hair, and color, and by equalizing low level image statistics. After the presentation phase, participants indicated to what extent the robots appeared uncanny and humanlike, and whether they could imagine social interaction with the robots in real life situations. The results show that robots rated as nearly humanlike scored higher on uncanniness, scored lower on imagined social interaction, evoked weaker pupil dilations, and their emotional expressions were more difficult to recognize. Pupils dilated most strongly to negative expressions and the pattern of pupil responses across emotions was highly similar between robot and human stimuli. These results highlight the usefulness of pupillometry in emotion studies and robot design by confirming the uncanny valley and media equation hypotheses.

## Highlights

1. The uncanny valley was confirmed with a subjective questionnaire about robots.
2. The pupil dilated less to emotional expressions of uncanny robots.
3. The pupil responded similarly to robotic and human emotional facial expressions.
4. Pupillometry thus confirms the uncanny valley and media equation hypotheses.

## Introduction

Developments in material, electronic, and computer sciences have advanced substantially over the last few decades. Many tasks that were previously performed by humans are now taken over by intelligent robots. One of the first robot with a simple “brain” was the *Machina speculatrix*, an electromechanical machine designed by W. Grey Walter that was capable of permuting relatively complex, autonomous behaviors with simple electrical simulations (Walter, 1950). Further developments in robotics progressed to current state-of-the-art humanoids that can walk, recognize auditory commands, talk back to provide requested information, and interact socially with humans (Dautenhahn, 2007; Krach et al., 2008). Contemporary social robots can improve the mood of elderly (Wada et al., 2005) or even trigger socially interactive behavior in persons with a diagnosis of autism (Dautenhahn and Werry, 2004; Damm et al., 2013). At some point robots may possess diverse social behavioral skills (Pfeifer et al., 2007), such as being able to hold a conversation. One central assumption in robotic design is that the more humanlike so-called “social” robots appear, the more the user will expect the robot to behave like a human being (Duffy and Joue, 2004), and to engage into social interactions with them. However, robots that look a lot but not quite like humans appear odd and eerie (Tinwell et al., 2011). This phenomenon is often explained in the context of the uncanny valley hypothesis (Mori, 1970), a theoretical assumption that the level of eeriness is explained by an observer’s unfamiliarity with humanlike robotic faces. Robots that appear almost humanlike but just not enough to evoke feelings of familiarity may instead appear like an eerie creature (Seyama and Nagayama, 2007; Ho and MacDorman, 2010; Mori et al., 2012; Piwek et al., 2014). Although it is interesting to discuss whether familiarity or another construct underlies the uncanny valley (e.g., Brenton et al., 2005; Kätsyri et al., 2015), we here only focus on how the uncanny valley can be measured.

**FIGURE 1:**
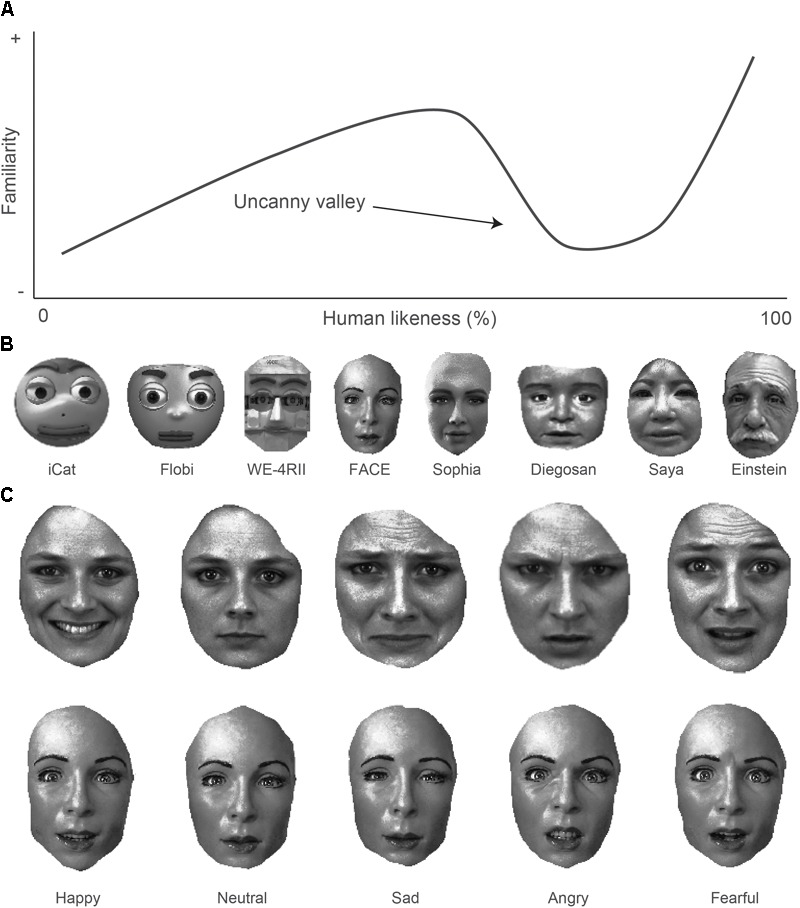
The uncanny valley and stimuli. **(A)** Feeling of familiarity (canniness) as a function of human likeness of robots (Mori, 1970). The dip in the non-linear function indicates the uncanny valley. **(B)** Robot faces ordered along the dimension of human likeness. **(C)** Example of emotional expressions by a human (top row) and FACE robot character (bottom row).

Although the evidence is limited (Gee et al., 2005), several studies have examined qualitative uncanniness ratings about robotic or virtual characters with varying realistic and naturalistic appearances and found a pattern in line with the uncanny valley as depicted in **Figure 1A** (e.g., Dill et al., 2012; Piwek et al., 2014; Mathur and Reichling, 2016; Strait et al., 2017). These findings have had considerable impact on the field of robotic design as they mean that near human-looking robots may evoke undesired, negative, affective responses (Vinayagamoorthy et al., 2005). As often happens in science, increasing popularity for an impactful hypothesis also leads to critical studies, disputing the existence or validity of the uncanny valley (Bartneck et al., 2007, 2009; Hanson, 2005; MacDorman, 2006; Pollick, 2009; Tinwell and Grimshaw, 2009; Zlotowski et al., 2013) for diverging reasons, including (1) a lack of emperical evidence, (2) a lack of strict overlap between reported uncanny ratings and the originally proposed valley function, and (3) indications that other factors (e.g., aesthetics) than familiarity underlie uncanny ratings of near-humanlike robots. However, a potential explanation for why some studies have failed to find a non-linear, valley-like relationship between familiarity (or canniness) and robotic human likeness is that (i) they did not control for aesthetically confounding low and high-level image properties such as image luminance and emotional expressions of the robots, (ii) tested the presence of the uncanny valley with only a small set of robotic stimuli, or (iii) varied human likeness within a character (e.g., morphing) – a procedure that produces rather unrealistic characters in general. Nevertheless, the current literature has so far reached no consensus on whether the uncanny valley is a relevant phenomenon for the field of human–robot interaction.

A related question to this issue is whether we can accurately measure whether or not a humanlike robot falls within the uncanny valley when so many factors affect the experience of familiarity and canniness. While it is obvious that the qualitative assessment of observers’ opinions about a robot’s appearance (e.g., uncanniness ratings) may shed light on this, it is also known that subjective impressions can be unreliable (Campbell, 1958). Quantitative measures have been proposed as alternatives (Wang, 2006), such as the use physiological responses or behavioral responses during robot interaction as an indication of the naturalness of the appearance or actions of robots and virtual avatars (Mower et al., 2007; Mohammad and Nishida, 2010; Weistroffer et al., 2013; Zanchettin et al., 2013; Strait and Scheutz, 2014; Strait et al., 2015). Electroencephalography (EEG) is also useful as a quantitative and neuroscientific measure of robotic facial processing (Dubal et al., 2010). The N170 component tends to reflect the function of the uncanny valley (Schindler et al., 2017). Specifically, the observers’ amplitudes of the N170 component consisted of a U-shaped pattern for which robots with intermediate realistic designs had weaker negativity at around 170 ms in relation to the preceding positive P100 component.

Here we aimed to use a novel, objective, physiological measure of uncanniness, namely pupil size. The amplitude of pupil responses is known to vary depending on the appearance of faces (Geangu et al., 2011; Laeng et al., 2013), naturalness of images (Naber and Nakayama, 2013), and attractiveness of objects (Wiseman and Watt, 2010; Leknes et al., 2012). The pupil also becomes relatively larger when observers view a familiar as compared to an unfamiliar object or scene (e.g., Võ et al., 2008; Kafkas and Montaldi, 2011; Naber et al., 2013b). This suggests that the pupil may serve as an objective measure of familiarity and thus the uncanny valley.

In addition to familiarity, emotions also play an important role in establishing satisfactory interactions (Schutte et al., 2001; Lazarus, 2006). In fact, the media equation hypothesis suggests that humans react socially to computers (Moon et al., 1997; Nass and Moon, 2000), virtual characters (Hoffmann et al., 2009; von der Pütten et al., 2010), and robots (Yan et al., 2004; Bethel, 2009). Indeed, physiological arousal increases in humans when viewing robots or virtual agents that experience a traumatic event (Slater et al., 2006; Rosenthal-von der Pütten et al., 2013a; Menne and Schwab, 2017), indicating a socially driven emotional response that is typical for human–human interaction. In a similar vein as the uncanny valley hypothesis, evidence for the media equation hypothesis in emotional human–robot interaction is limited. The development of robots that can display facial, emotional expression is becoming more popular (Breazeal, 2003; Zecca et al., 2004; van Breemen et al., 2005; Hashimoto et al., 2006; Hegel et al., 2010; Becker-Asano and Ishiguro, 2011; Mazzei et al., 2012; Damm et al., 2013; Salvador et al., 2015), but it is not entirely clear whether such robotic features have benefits (Bruce et al., 2002, but see Leite et al., 2008), especially when considering that humans may not process and react to robotic emotions as compared to human emotions. To investigate whether physiological responses to emotional situations are comparable between robots and humans, we include robotic, emotional, facial expressions in the current experimental design.

Evidence from EEG studies suggest that the visual system responses similar to robotic faces as human faces (Dubal et al., 2010; Schindler et al., 2017). Pupillometry would again be a useful alternative measure for a thorough investigation of the media equation hypothesis, since pupil size is especially sensitive to the emotional content of facial expressions. More pupil dilation is evoked after the observation of arousing, negative emotional expressions in faces as compared to less arousing, neutral, and positive expressions (Geangu et al., 2011; Burkhouse et al., 2015).

The temporal dynamics of the pupillary response thus provide a rich marker for ongoing cognitive and emotional processes that might be indicative for how human and robotic faces are perceived. Although many other aspects may play a role in human–robot interaction and for the potential acceptance of social robots into the daily lives of humans, we here aim to contribute to the broad and multidisciplinary investigation into the perceptual component of interactions with robots by assessing subjective opinions and pupillary responses to robots that can express several emotions.

In the present study we investigate how the sensory processing of facial emotional expressions may give way to understanding how participants experience robots. We hypothesize that when participants process robotic emotions just like human emotions, the pupil should respond similarly to emotional expressions of robots and humans. Moreover, if observers experience robotic expressions as special, awkward, uninteresting, unattractive, ugly, and thus uncanny, their pupils should respond less vigorously. To do so, we investigate the pattern of pupillary responses to a variety of facial emotional expressions, displayed by human agents, or displayed by social robots that vary in human likeness and eeriness, while strictly controlling for low-level (i.e., luminance, contrast, color) and high-level (i.e., image resolution; head orientation; hairstyle) image statistics of the faces (see **Figures 1B,C**).

## Materials and Methods

### Participants

Forty individuals participated in the experiment (mean age: 21.17, SD: 1.34 years, 30 females) in which they observed pictures of robotic and human faces that expressed a variety of emotions. Such sample sizes are more than sufficient to find differences between emotional conditions in within-design pupillometry studies (Naber et al., 2012). All participants had normal or corrected-to-normal vision, were naïve to the purpose of the experiment, and gave informed consent before the experiment. All participants were Dutch university students recruited through social networks, or with flyers and posters. This study was carried out in accordance with the recommendations of the local ethics commission of the Utrecht University. The protocol was approved by the local ethics commission (FETC) of the Utrecht University. All subjects gave written informed consent in accordance with the Declaration of Helsinki.

### Apparatus

The participant’s head was supported by a chin- and forehead-rest to enable gaze-tracking and pupil size recordings. Pupillometry data was obtained with an infrared sensitive camera (EyeLink 2000, SR Research, Osgoode, ON, Canada) that tracks gaze and pupil size at a rate of 1000 Hz. The eye-tracker was calibrated at the start of each experiment using a 13-point calibration grid. The video stimuli were presented on an LED Asus ROG swift monitor (AsusTek Computer Inc., Taipei, Taiwan), at a viewing distance of 55 cm. The refresh rate of the screen was 60 Hz and the resolution was 2560 pixels × 1440 pixels. Blank screens and backgrounds surrounding the images were gray. Stimuli were generated on an Optiplex 755 DELL computer, using Matlab (Mathworks, Natick, MA, United States), the Psychophysics toolbox (Brainard, 1997), and EyeLink toolbox extensions (Cornelissen et al., 2002).

### Stimuli and Procedure

The experiment was designed to measure physiological reactions to and appearance ratings about the presentation of pictures of robotic and human faces that expressed a variety of emotions. The first block consisted of the presentation of human and robot pictures while pupillary responses were recorded with the eye-tracker. Pictures of humans were collected from the KDEF database and displayed five possible emotions: happy, neutral, sad, angry, and fearful (Lundqvist et al., 1998). Pictures of robots were found on the world wide web and displayed the same emotions as the humans. We incorporated only robots in the stimulus set that were physical (i.e., not virtual but robots made out of real materials). For reliable correlational comparisons of pupil responses to humans versus robots across emotions (see section “Analysis” below), we incorporated only robots for which we could find pictures of at least four of the five human emotional expressions (see **Table 1**). The final robot picture set consisted of eight robots that varied along the dimension of human likeness, including three non-humanlike robots called iCat (van Breemen et al., 2005), Flobi (Hegel et al., 2010; Lutkebohle et al., 2010), WE-4RII (Zecca et al., 2004), and five humanlike robots called FACE (Mazzei et al., 2012), Sophia, Diego-san, Einstein (Hanson Robotics) aka Albert HUBO (Oh et al., 2006), and Saya (Hashimoto et al., 2006). All faces faced directly the observers. Facial hair and backgrounds were removed by cutting out only the skin part of the faces. Colors were removed and all robotic and human pictures were then equalized in luminance and luminance contrast through histogram equalization, and equalized in surface size (50000 non-transparent pixels; 878 by 717 pixels). The controls described above ensured that several confounding factors could not additionally influence pupil size, aesthetics, and other factors unrelated to canniness and emotions (Naber et al., 2012). Examples of the resulting robot stimuli are shown in **Figure 1B**. An example of each emotional expression for the robot FACE and for a human character are shown in **Figure 1C**. See Supplementary Figure S1 in the online supplemental materials for the emotional expressions per robot.

**Table 1:** Available emotions per robot (o = present, x = absent).

Robot	Happy	Neutral	Sad	Angry	Fearful
iCat	o	o	x	o	o
Flobi	o	o	o	o	o
WE-4RII	o	o	o	o	o
FACE	o	o	o	o	o
Sophia	o	o	o	o	o
Diegosan	o	o	o	x	o
Saya	o	o	o	o	x
Einstein	o	o	x	o	o

In the first block, pictures were shown for 3 s, followed by a response from the participants. We instructed participants to classify the emotion expressed by the face in the picture. An image of a number pad with corresponding expressions per button was shown after each picture (2 = sad, 4 = angry, 5 = neutral, 6 = fearful, 8 = happy). Participants could select one of the expressions by pressing the corresponding button on a keyboard, and were given maximally 2 s of time to respond. The following trial was initiated with the press of a button and the picture was preceded by the presentation of fixation dot for a random duration selected from a range between 0.5 and 1.5 s. Participants received feedback in the first 12 trials about whether their correctly recognized the emotion (fixation turned green for 2 s), incorrectly recognized the emotion (fixation turned red), or were too late with their response (fixation turned blue). Participants were allowed to take a break half-way through the experiment. Each picture was shown three times, appearing randomly across the block. The first block consisted of a total of 228 trials (8 robots plus 8 humans, times 5 emotions, times 3 presentations = 240 trials, but one emotion was lacking for 4 robots, resulting in 240 – 4 robots times 3 presentations = 228 trials).

In the second block, each robot and human picture with a neutral expression was shown again for 3 s, followed by four separate screens with each a different question about the robots’ appearances (e.g., attractiveness; see Q1–Q4 in **Table 2**) requiring a rating on a unidimensional scale from 0 to 100. The participants could use a computer mouse to indicate on the scale to what degree the robot scored on the scale naturalness, human looking, attractiveness, and eeriness per screen. The third block was the same as the second block but this time only the neutral robot faces were shown again and participants were asked to indicate how comfortable they would find it to interact with the robot in a variety of futuristic contexts (e.g., to assist during studying; see Q5–Q9 in **Table 2**). We added these questions to generalize the uncanny valley to other contexts, specifically to investigate whether the uncanny valley may also apply in a more interactive rather than mere observational situation.

**Table 2:** Questionnaire items.

#	Tag	Question	Rating scale
Q1	Naturalness	How natural is the robot’s appearance?	0 = unnatural, 100 = natural
Q2	Human looking	How human is the robot’s appearance?	0 = not human, 100 = human
Q3	Attractiveness	How attractive is the robot’s appearance?	0 = unattractive, 100 = attractive
Q4	Eeriness	How eerie is the robot’s appearance?	0 = eerie, 100 = reassuring
		To what degree would you feel comfortable when the robot would …	
Q5	House keeping	… help you with house keeping	0 = uncomfortable, 100 = comfortable
Q6	Information	… provide you with daily information such as the local news	0 = uncomfortable, 100 = comfortable
Q7	Conversation	… be a person who you could talk to	0 = uncomfortable, 100 = comfortable
Q8	Study help	… help you during studying	0 = uncomfortable, 100 = comfortable
Q9	Finance	… do your finances and administration	0 = uncomfortable, 100 = comfortable

### Analysis

A principal components factor analysis with direct oblimin rotation and Kaiser normalization was performed to investigate common underlying factors across multiple questions explaining the variance in subjective ratings. Factors with eigenvalues higher than 1 were used for further analysis. One-way repeated measures ANOVA with the predicting factor of robot character was performed on each questionnaire factor from the principle component analysis.

For the pupil analyses, we first removed trials in which emotions were not recognized (22.03% ± 4.34% of all trials) or recognized too late (>2 s; 1.34% ± 1.33% of all trials). Incorrect trials were removed because it is known from previous pupillometric investigations that subjective miscategorization suppresses differences in pupil responses between conditions (e.g., Naber et al., 2013b). Nonetheless, the inclusion of all trials or the treatment of subjectively recognized emotions as ground truth (i.e., an emotional expression objectively presented as sad but subjectively recognized as fearful was treated as objectively fearful) did not produce qualitatively different results. We additionally interpolated blink periods in the pupil traces with cubic spline fits. To enable comparison across emotions, we then baseline corrected pupil size by subtracting pupil size at the moment of face onset. To enable comparison across participants, pupil size was converted to *z*-scores by dividing each pupil trace by the standard deviation of pupil size during all trials.

To compare the extent of pupil dilation across emotional expressions, average pupil size was calculated between 1 and 3 s, that is around peak pupil dilation and well after an initial pupillary light response. A two-way repeated measures ANOVA with predicting factors of agent (human vs. robots) and emotion was performed on the average pupil dilation. *Post hoc* statistical tests consisted of two-sided, paired sample student *t*-tests to compare which emotional conditions differed significantly. Pearson’s correlation coefficient was used to calculate the degree of correlation between pupillary responses to robots versus human emotions.

To scrutinize which factor explains most variance in uncanniness ratings across robot characters, general linear models were calculated. Input to this model were data based on the measures per participant and per robot character (8 times 40 data points per predictor or dependent variable).

## Results

### Uncanny Valley

We designed an experiment in which 40 human participants viewed faces while their pupil responses were recorded with an eye-tracker. Their task was to indicate which emotion was observed [see subsequently, participants rated each human and robotic face with neutral expressions on nine dimensions (**Table 2**).

Our first aim was to replicate the uncanny valley effect in the subjective scoring of the robot stimuli, emotion recognition scores, and pupil size. To inspect these effects, we first needed to know which factors were present in the scorings of the 9 questions about robots. Three factors emerged from the factor analysis. Question 1 (naturalness; loading: 0.964) and question 2 (human looking; loading: 0.977) were combined in the factor that we call *human likeness* (eigenvalue 1.165). Question 3 (attractiveness; loading: 0.856) and question 4 (eeriness; loading: 0.837) together measured the second factor that we call *canniness* (eigenvalue = 2.212). Questions 5 to 9 (loading: 0.875, 0.919, 0.843, 0.825, 0.744, respectively) were combined as the third factor *interaction* (eigenvalue = 3.833). These three factors accounted for 80% of the variance.

**FIGURE 2:**
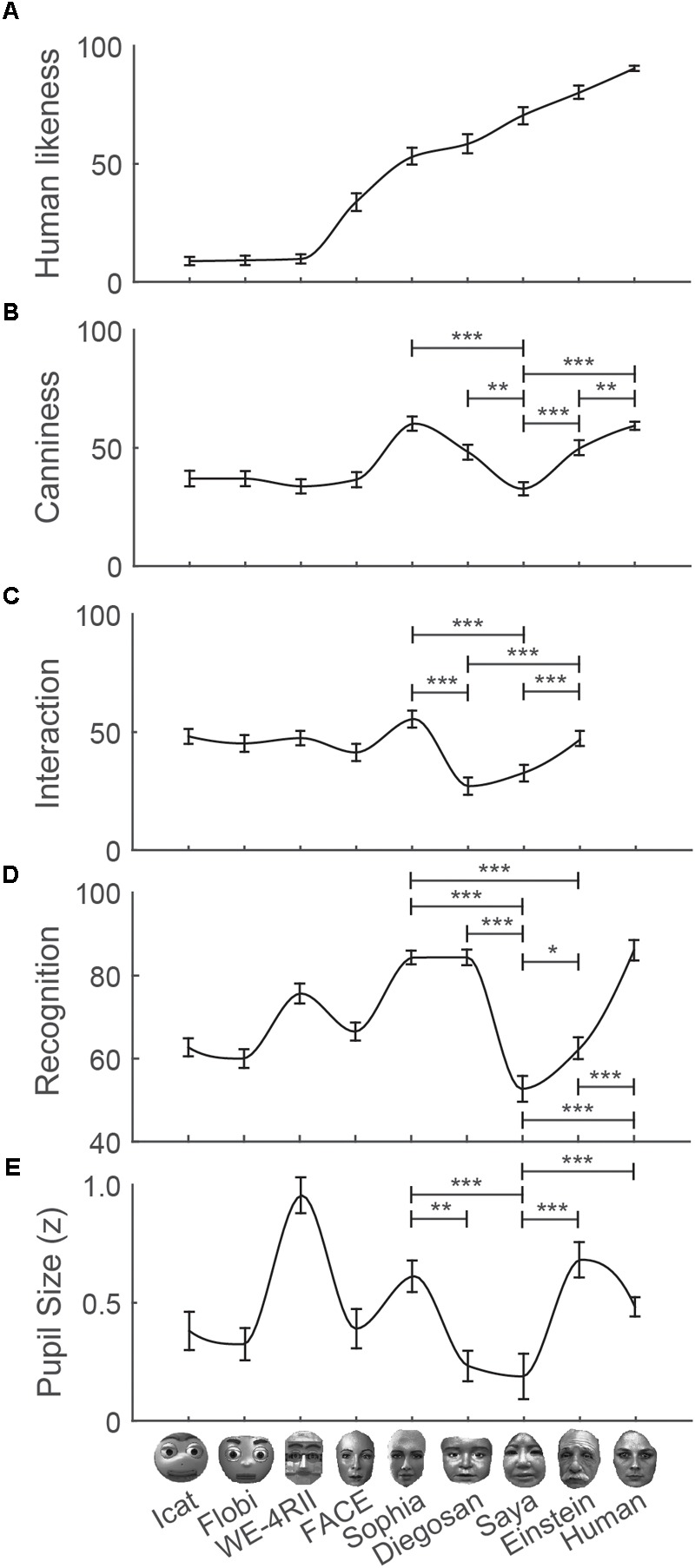
The uncanny valley confirmed: average scores with standard errors on a variety of measures. **(A)** Human likeness across participants, **(B)** uncanniness, **(C)** comfortability with robotic applications, **(D)** emotion recognition performance, and **(E)** average pupil size between 1 and 3 s after stimulus onset per robot character. The robot characters are ordered by scores on human likeness and the last character (outmost right) shows the average score pooled across all human characters. The most relevant statistical comparisons between robots are indicated with asterisks (^∗^*p* < 0.05, ^∗∗^*p* < 0.01, ^∗∗∗^*p* < 0.001).

We first assessed whether the robot characters varied in the factor human likeness. Indeed, we found a main effect of human likeness (**Figure 2A**; for ANOVA results, see Supplementary Table S1 in online Appendix; for *post hoc* comparisons, see online Supplementary Table S2). More importantly, we examined whether the other factors from the questionnaire (canniness and interaction) and other variables (i.e., emotion recognition performance and pupil size) varied along the dimension of human likeness, showing a decrease in canniness (i.e., the uncanny valley) for robots scoring high though lower than 100% on the dimension of human likeness. **Figures 2B–E** shows the presence of valleys around the robots Diegosan, Saya, and Einstein for canniness, interaction, emotion recognition scores, and pupil size. Overall these robots scored significantly lower on these factors than the robots adjacent to them on the dimension of human likeness. Despite small variations in the location of the trough of the uncanny valley function, the overall pattern of statistical comparisons support the presence of an uncanny valley in each factor (for *post hoc* comparisons, see online Supplementary Tables S3–S6; for uncanny valley patterns per emotion, see Supplementary Figures S2A,B; for patterns of correctly versus incorrectly recognized emotions, see Supplementary Figure S2C). The only robot that did not follow the uncanny valley pattern was the non-humanlike robot WE-4RII that evoked remarkably large pupils as compared to other robots.

To further investigate which factors underlie the uncanny valley, we calculated a general linear model with the predictors recognition scores and average pupil size to explain variance in canniness across the robot characters. The resulting, unstandardized betas indicated that recognition scores (*B* = 17.05, *p* = 0.012) but not pupil size (*B* = -0.95, *p* = 0.723) explained variance in canniness ratings. Additional general linear models that predicted either recognition scores (Canniness: *B* = 0.001, *p* = 0.012; Pupil size: *B* = 0.09, *p* < 0.001) or pupil size (Canniness: *B* < 0.001, *p* = 0.723; Recognition: *B* = 0.611, *p* < 0.001) suggested that emotion recognition scores is partially responsible for both uncanny ratings [*r*(318) = 0.14, *p* = 0.013] and weaker pupil dilations [*r*(318) = 0.24, *p* < 0.001] of the eerie robots that fall within the uncanny valley but that uncanny ratings do not relate to weaker pupil dilations [*r*(318) = 0.01, *p* = 0.804]. However, the relationships reported above are weak. This withholds us from concluding that the uncanny valley was merely determined by recognition scores.

### Media Equation

**FIGURE 3:**
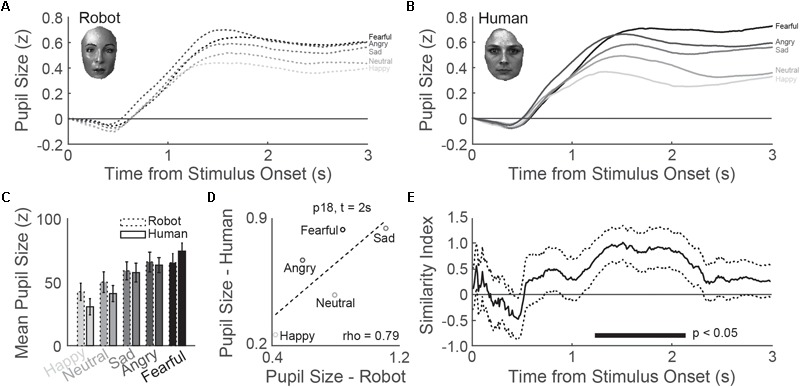
Media equation confirmed: pupil response similarity between robot and human emotions. **(A,B)** Average pupil responses to robot faces across participants were comparable to human faces. **(C)** Average pupil dilation between 1 and 3 s was larger for robot and human emotions with a negative valence. **(D)** Example correlation between average pupil size for human (*y*-axis) versus robotic (*x*-axis) faces across emotional expressions. **(E)** Similarity index as a function of time after stimulus onset, based on the correlation between robot and human average pupil size across emotions (see **D**), indicated significant overlap in pupil response profiles between ∼1 and 3 s after emotion onset. The horizontal bar at the bottom indicates at which time points the similarity index was significantly larger than zero. The dotted lines indicate the standard error of the mean.

Our second aim was to investigate whether the participants’ pupils responded similar to the onset of emotional expressions of robot and human stimuli. As shown in **Figures 3A,B**, pupil size responded to the onset of robot and human stimuli with a gradual dilation over time that reached peak dilatation around one and a half second after stimulus onset. The average pupil dilation between 1 and 3 s after picture onset varied significantly across emotional expression (for ANOVA results, see online Supplementary Table S7; for *post hoc* comparisons across emotions, see online Supplementary Table S8), reaching larger dilations for emotions with negative valences (sadness, fearful, and anger) as compared to positive (i.e., happy) and neutral valences (**Figure 3C**). Human and robotic expressions did not evoke different patterns of pupil dilations and the interaction between agent and emotion was not significant. These results demonstrate that the pupil responds differently to each emotional expression but that the pattern of pupil dilation across emotions is comparable between humans and robots.

The same analysis as above was performed for emotion recognition scores. Robotic emotional expressions were more difficult to recognize in general, and recognition scores across emotions showed dissimilar patterns between humans and robots (for statistics, see online Supplementary Tables S9, S10; for emotion confusion matrices, see Supplementary Figures S3, S4). This means that the pupil responded similarly to humans and robots across emotions despite differences in recognition scores across emotions.

Next, we examined to what degree pupil responses were comparable between robot and humans by calculating correlations between average pupil responses to robots and humans. Independent of emotion, average pupil size correlated quite well between human and robot conditions across subjects [*r*(38) = 0.77, *p* < 0.001]. Correlations across emotional expressions were also significant. **Figure 3D** displays an example of a correlation of average robot versus human pupillary responses calculated at 2 s after emotion onset across emotions for a single participant. We calculated this correlation for each subject and for each time point from stimulus onset (for the average correlation across all subjects, see Supplementary Figure S5 in online Appendix). The correlation became significant after approximately 1.2 s after stimulus onset, reaching peak correlation around 1.5 s. Although peak correlation was weak (rho = 0.30) it should be taken into account that these correlations will not get higher than a certain threshold because of considerable noise in the data (i.e., only 4–5 data points are correlated). For the interpretation as to whether a rho of 0.30 indicates a relatively low or high similarity in pupil patterns, we needed to know how strong these correlations could get in theory. The correlations of pupil response patterns between human faces would serve as a proper basis for a theoretical ceiling correlation. As such, we calculated the average correlation per time points across all possible comparisons between any two human characters (i.e., 28 comparisons in total). The original correlation traces (see Supplementary Figure S5) were subsequently compared to the ceiling correlation traces by calculating a similarity index per participant, that is the original correlations robot versus human divided by the ceiling correlations. The average of this pupil similarity index across participants is shown in **Figure 3E**. A full similarity index was reached around 1.5 s after picture onset and remained stable for another 0.5 s. Note that this period corresponds to the period at which pupil dilation peaks in response to the emotion (**Figures 3A,B**), that is the time point at which the pupil is most sensitive to and reflects best emotion processing. In sum, the pattern of pupil responses to robotic emotions were just as similar to the pattern of pupil responses to human emotions as pupil responses compared between humans. These results indicate that the visual processing and resulting physiological responses are similar for human and robotic emotional expressions.

## Discussion

We investigated the recognition of emotional facial expressions displayed by robots and humans and measured pupillary responses as objective markers of visuo-emotional processing by the nervous system. We found that recognition scores and pupil size per character followed the pattern of the uncanny valley. Robotic faces that fall within the uncanny valley because they look almost like humans but not quite right, were more difficult to recognize and evoked weaker pupil dilations. This finding concurs with previous research that used brain activity instead of physiological measures as a marker of eeriness (Dubal et al., 2010; Schindler et al., 2017).

We additionally found that pupils dilated most when participants viewed emotional expressions with a negative valence as compared to a positive or neutral valence. These findings are in line with existing literature on pupil dilations to the presentations of emotional expressions and stimuli (Bradley et al., 2008; Geangu et al., 2011; Al-Omar et al., 2013; Laeng et al., 2013; Burkhouse et al., 2015), but it is the first time that this pattern of responses was found for robotic emotional expressions.

The fact that the similarity in pupil response patterns between human and robot faces (between agent types) reached the same level of similarity of comparisons between human faces (within agent type) suggests that, the peripheral nervous system, and the brain regions that innervate it, share a common underlying mechanism for the processing of robotic and human emotional expressions. Hence, physiology suggests that robot emotions are processed as humanlike emotions, a finding that confirms the media equation hypothesis (Moon et al., 1997). Previous EEG studies found indications for the media equation hypothesis (Dubal et al., 2010; Schindler et al., 2017), and the current study it is the first to confirm the hypothesis and quantify the degree of similarity.

Our study also confirms the crucial role of human likeness and emotion recognition during human–robot perception, even after controlling for factors that may have influenced aesthetical factors. Although it remains unclear what the uncanny valley truly reflects (aesthetics, problems with emotion recognition, etcetera) and what underlies media equation, we deem it here most important that recognition performance and pupil size can be used as indicators of uncanny and emotional feelings for robotic characters. The usefulness of the pupil response as a neurophysiological marker of uncanniness and emotion recognition opens up a potential application of pupillometry. For example, the current experimental design and pupillometry could be used as a visuo-emotional version of the Turing test to assess whether a robot’s emotional expressions and appearance have passed the uncanny valley and are recognized as truly human rather than robotic. Pupil size may thus indicate whether observers treat a robot, either consciously or unconsciously, as familiar and reassuring.

A limitation of the current study is that we did not measure uncanniness and human likeness ratings per robotic emotional expression. Certain negative, withdrawal emotions can affect the uncanny appearance of virtual characters (Ho et al., 2008; Tinwell et al., 2011). Here, ratings of uncanniness were only assessed for robots with neutral expressions to prevent order effects (i.e., seeing an uncanny, fearful expression may affect the subsequent rating of a canny, neutral expression). Although out of the scope of the current experiment, future studies may want to examine whether the uncanny valley is strengthened or weakened as a function of the depicted emotional expression. Another limitation is that it is difficult to control for all factors that may affect pupil size and subjective experiences. For example, attention, aesthetics and to what degree observed persons are trusted can alter the amplitude of dilatory pupil responses (e.g., Wiseman and Watt, 2010; Leknes et al., 2012; Naber et al., 2013a; Kret et al., 2015) or subjective canniness ratings (e.g., Hanson, 2005). The observation of strange though comical expressions (Blow et al., 2006) may also affect pupil dilation (e.g., a fearful expression of WE-4RII may instead appear funny). Lastly, the current experiment was relatively passive and we presented still images of robots. The findings may not generalize to more active conditions in which an interaction is required or dynamically moving robots are shown (Mohammad and Nishida, 2010, but see Piwek et al., 2014). Such factors related to appearance and ecological validity need to be considered in future research. Nevertheless, we find it relevant to denote that, to our knowledge, this is the first study on the uncanny valley and media equation hypotheses that strictly controlled for confounding low-level image statistics such as luminance, contrast, and color. Differences in these factors across robotic faces could potentially be the basis for aesthetical evaluation, a factor that has been proposed as an alternative for familiarity to underlie the uncanny valley (Hanson, 2005).

Although humans respond to robot and human emotions alike, does this imply that humans have emotions and affections for robots that are undistinguishable from emotions and affections for humans? In line with the media equation hypothesis, it is tempting to speculate that the here reported similarity of physiological responses to robot and human emotional expressions points at a potential resonance between humans and robots at the level of emotion recognition. Our findings further confirm previous results from human–robot interaction studies showing affectionate responses to robots by humans (for a nice overview of the literature on emotions and human–robot interaction, see Rosenthal-von der Pütten et al., 2013a; Menne and Schwab, 2017). For example, an elegant study measured heart rate and skin conductance to show that humans become physiologically aroused when they view videos of a robot being tortured (Rosenthal-von der Pütten et al., 2013a). Besides the processing of such emotional situations, it is also important to investigate physiological responses to robots that produce emotional reactions themselves, a research area that has received, in our opinion, not enough attention. An other research topic that is interesting to pursue scientifically is the role of social intelligence, such as theory of mind (Krach et al., 2008), or automatic and unconscious perception-action coupling, such as imitation (Oztop et al., 2005; Press et al., 2005; Sciutti et al., 2012; Hofree et al., 2015) in the context of negative (Naber et al., 2013c, 2016) and more realistic situations (Mower et al., 2007; Mohammad and Nishida, 2010).

We wonder in which other contexts than visuo-emotional processing humans may not respond similarly to robots as they normally do to humans. Here we only looked at relatively low-level responses to visual content. Neuroscientific evidence suggests that, depending on the task, some brain areas respond weaker to observed robots than to humans (Gazzola et al., 2007; Saygin et al., 2011; Rosenthal-von der Pütten et al., 2013b). Especially in real-life scenarios, the context is more complex, and interaction relies on other, more cognitive mechanisms, such as decision-making and social values. For example when people receive positive or negative feedback about their personality or play a social decision-making game, brain areas involved in social cognition also tend to respond less strong during interaction with a computer rather than a human (Kircher et al., 2009; Chaminade et al., 2012; Schindler et al., 2015; Schindler and Kissler, 2016). It is possible that such (neuro)physiological differences are due to a lacking experience with artificial intelligence, less appraisal by humans for a computer-generated opinion, or devaluation of the consequences of an artificial and random interaction produced by code – state-of-the-art artificial social intelligence is far from human like – rather than due to a lacking affinity with artificial intelligence.

We are also curious how distinct the processing of robotic emotional features can be from human standards and still elicit affectionate responses. The visual distinctiveness and aesthetical appearance of robots may play an additional role in the uncanny valley (Zell et al., 2015). Indeed, the robot WE-4RII looks quite distinct (oddball) and increases the eye’s pupil considerably more than other robots. Although this interpretation is speculative, it is in line with the observation that pupil dilation to the human characters, which all looked alike, was weaker than to the pupil dilation to Sophia and Einstein, which differed more in facial features and may have appeared more like an oddball than the humans.

Taking into account the likeliness that robots will soon be part of many people’s lives, we consider the exploration of the consequences of commonalities and differences between robots and humans in the near future as highly exciting and relevant in robotic and social, affective, behavioral, and biological sciences.

## Author Contributions

AR, MvD, and MN designed the experiments. MvD and AR collected the data. MN programmed the experiments and all authors contributed to the data analysis. MN wrote the first drafts of the paper and all other authors helped finalizing the final paper.

## Conflict of Interest Statement

The authors declare that the research was conducted in the absence of any commercial or financial relationships that could be construed as a potential conflict of interest. The reviewer RK and handling Editor declared their shared affiliation.
