# Supplementary material for: Pupillary Responses to Robotic and Human Emotions: The Uncanny Valley and Media Equation Confirmed
Source: Front Psychol. 2018 May 23;9:774. doi: 10.3389/fpsyg.2018.00774 (PMC5974161; doi:10.3389/fpsyg.2018.00774)
Supplement: Supplementary file 1 [file Data_Sheet_1.docx]

# Appendix

**Validation of robotic emotional expressions**

**
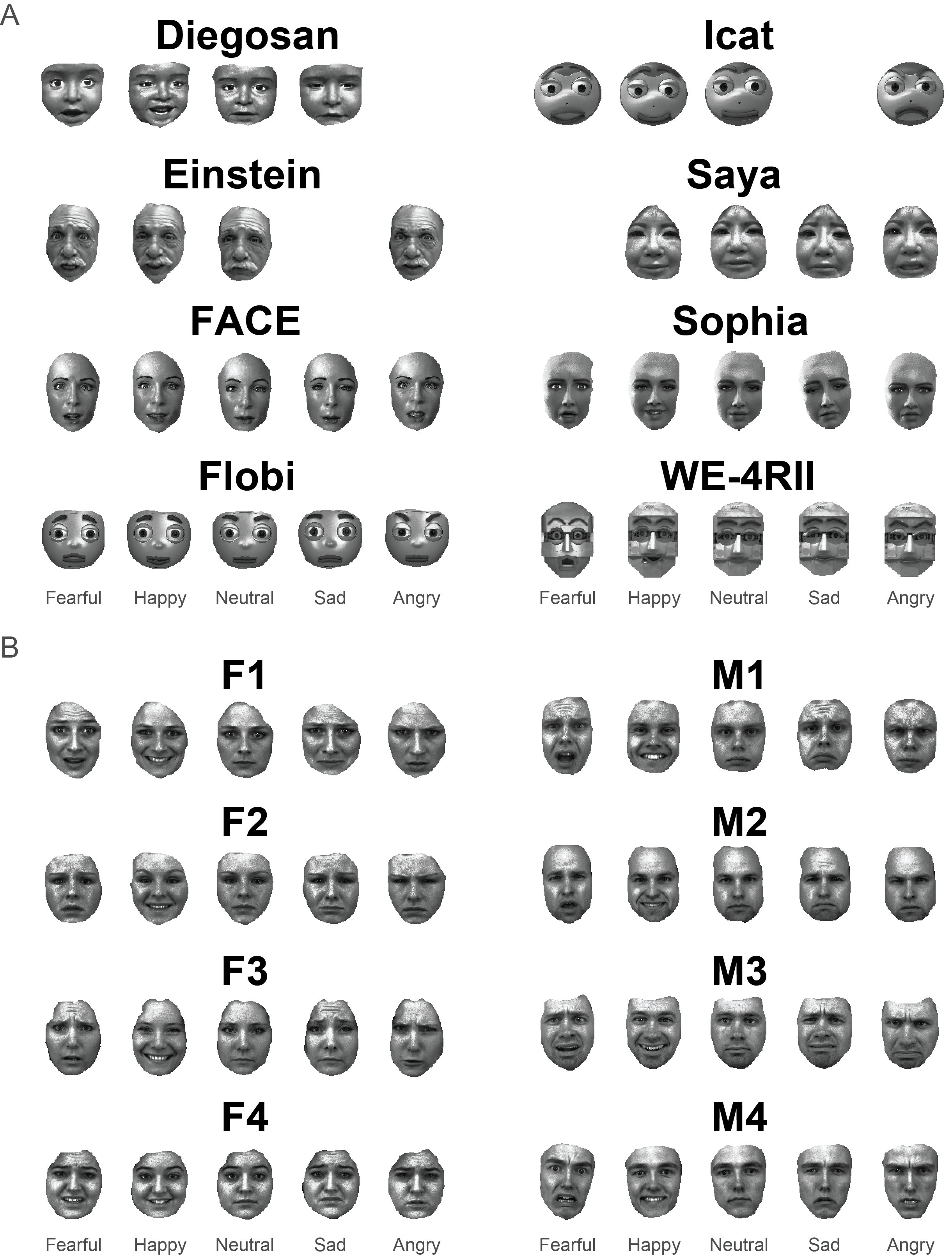
**

**Figure S1.** Emotional expressions per robot (A) and human (B) character.

**Analysis of factor scorings across robot characters**

*Table S1. Results of ANOVAs per factor with robot characters as independent variable*

| **Dependent variable (factor*)*** | ***F*-statistic** | ***df*** | ***p*-value** |
| --- | --- | --- | --- |
| Human likeness | 117.092 | (7,39) | <0.001 |
| Canniness | 12.178 | (7,39) | <0.001 |
| Interaction | 11.910 | (7,39) | <0.001 |
| Recognition | 25.968 | (7,39) | <0.001 |
| Pupil Size | 16.138 | (7,39) | <0.001 |

*Table S2. Post-hoc comparisons of factor human likeness across robot and human faces (t-value, p-value)*

|  | Flobi | WE-4RII | FACE | Sophia | Diegosan | Saya | Einstein | Human |
| --- | --- | --- | --- | --- | --- | --- | --- | --- |
| iCat | 0.21, 0.838 | 0.51, 0.614 | 7.46, 0.000 | 11.88, 0.000 | 11.45, 0.000 | 16.14, 0.000 | 25.02, 0.000 | 37.81, 0.000 |
| Flobi |  | 0.36, 0.722 | 6.51, 0.000 | 10.51, 0.000 | 12.15, 0.000 | 16.12, 0.000 | 20.69, 0.000 | 36.09, 0.000 |
| WE4RII |  |  | 6.37, 0.000 | 10.52, 0.000 | 10.70, 0.000 | 15.56, 0.000 | 21.77, 0.000 | 32.96, 0.000 |
| FACE |  |  |  | 5.17, 0.000 | 5.09,  0.000 | 7.34, 0.000 | 12.14, 0.000 | 14.44, 0.000 |
| Sophia |  |  |  |  | 1.07,  0.290 | 4.22, 0.000 | 6.67,  0.000 | 10.97, 0.000 |
| Diegosan |  |  |  |  |  | 2.94, 0.006 | 5.71,  0.000 | 8.60, 0.000 |
| Saya |  |  |  |  |  |  | 2.81,  0.008 | 5.99, 0.000 |
| Einstein |  |  |  |  |  |  |  | 3.76, 0.001 |

**Uncanny valley post-hoc analysis**

*Table S3. Post-hoc comparisons of factor canniness across robot and human faces (t-value, p-value). Table cell borders with the most relevant comparisons are dotted.*

|  | Flobi | WE-4RII | FACE | Sophia | Diegosan | Saya | Einstein | Human |
| --- | --- | --- | --- | --- | --- | --- | --- | --- |
| iCat | 0.00, 0.999 | 0.95, 0.349 | 0.13, 0.899 | 5.81, 0.000 | 2.47,  0.018 | 1.09, 0.284 | 3.49,  0.001 | 6.31, 0.000 |
| Flobi |  | 0.95, 0.350 | 0.13, 0.895 | 5.31, 0.000 | 2.63,  0.012 | 1.13, 0.267 | 3.02,  0.004 | 6.90, 0.000 |
| WE4RII |  |  | 0.75, 0.461 | 6.79, 0.000 | 3.14,  0.003 | 0.24, 0.815 | 4.27,  0.000 | 6.83, 0.000 |
| FACE |  |  |  | 6.25, 0.000 | 2.80,  0.008 | 1.15, 0.257 | 3.52,  0.001 | 6.53, 0.000 |
| Sophia |  |  |  |  | **2.94,**  **0.005** | **6.43, 0.000** | **2.30,**  **0.027** | 0.26, 0.795 |
| Diegosan |  |  |  |  |  | **4.39, 0.000** | 0.44,  0.664 | **4.02, 0.000** |
| Saya |  |  |  |  |  |  | **4.33,**  **0.000** | **10.33, 0.000** |
| Einstein |  |  |  |  |  |  |  | **3.09, 0.004** |

*Table S4. Post-hoc comparisons of factor application across robot faces (t-value, p-value). Table cell borders with the most relevant comparisons are dotted.*

|  | Flobi | WE-4RII | FACE | Sophia | Diegosan | Saya | Einstein |
| --- | --- | --- | --- | --- | --- | --- | --- |
| iCat | 1.25, 0.218 | 0.31, 0.758 | 1.77, 0.084 | -2.26, 0.030 | 5.20,  0.000 | 4.17, 0.000 | 0.22, 0.827 |
| Flobi |  | -0.80, 0.427 | 0.89, 0.377 | -2.29, 0.027 | 4.12,  0.000 | 2.98, 0.005 | -0.48, 0.636 |
| WE4RII |  |  | 1.61, 0.115 | -2.28, 0.028 | 5.29,  0.000 | 3.65, 0.001 | 0.03, 0.976 |
| FACE |  |  |  | -4.33, 0.000 | 3.78,  0.001 | 2.59, 0.013 | -1.62, 0.114 |
| Sophia |  |  |  |  | **6.29,**  **0.000** | **5.53, 0.000** | **2.10, 0.042** |
| Diegosan |  |  |  |  |  | -1.78, 0.082 | **-6.32, 0.000** |
| Saya |  |  |  |  |  |  | **-4.42, 0.000** |

*Table S5. Post-hoc comparisons of emotion recognition scores across robot and human faces (t-value, p-value). Table cell borders with the most relevant comparisons are dotted.*

|  | Flobi | WE-4RII | FACE | Sophia | Diegosan | Saya | Einstein | Human |
| --- | --- | --- | --- | --- | --- | --- | --- | --- |
| iCat | 0.83, 0.409 | 3.88, 0.000 | 1.32, 0.196 | 8.33, 0.000 | 7.42,  0.000 | 3.18, 0.003 | 0.06, 0.950 | 10.33, 0.000 |
| Flobi |  | 4.53, 0.000 | 2.00, 0.053 | 8.28, 0.000 | 7.63,  0.000 | 1.95, 0.059 | 0.65, 0.517 | 10.81, 0.000 |
| WE4RII |  |  | 3.13, 0.003 | 2.87, 0.007 | 2.77,  0.008 | 5.86, 0.000 | 4.44, 0.000 | 4.31, 0.000 |
| FACE |  |  |  | 6.28, 0.000 | 6.56,  0.000 | 4.03, 0.000 | 1.36, 0.182 | 8.43, 0.000 |
| Sophia |  |  |  |  | 0.02,  0.986 | **8.57, 0.000** | **7.14, 0.000** | 1.00, 0.324 |
| Diegosan |  |  |  |  |  | **9.34, 0.000** | **6.36, 0.000** | 0.99, 0.327 |
| Saya |  |  |  |  |  |  | **2.38, 0.022** | **10.60, 0.000** |
| Einstein |  |  |  |  |  |  |  | **8.48, 0.000** |

*Table S6. Post-hoc comparisons of average pupil size across robot and human faces (t-value, p-value). Table cell borders with the most relevant comparisons are dotted.*

|  | Flobi | WE-4RII | FACE | Sophia | Diegosan | Saya | Einstein | Human |
| --- | --- | --- | --- | --- | --- | --- | --- | --- |
| iCat | 0.61, 0.543 | -7.13, 0.000 | -0.09, 0.928 | -2.50, 0.017 | 1.61,  0.114 | 1.70, 0.096 | -3.14, 0.003 | -1.36, 0.183 |
| Flobi |  | -8.26, 0.000 | -0.62, 0.537 | -3.51, 0.001 | 1.09,  0.281 | 1.43, 0.162 | -4.05, 0.000 | -2.52, 0.016 |
| WE4RII |  |  | 6.10, 0.000 | 4.90, 0.000 | 10.41, 0.000 | 8.08, 0.000 | 3.43, 0.001 | 10.62, 0.000 |
| FACE |  |  |  | -3.09, 0.004 | 1.89,  0.066 | 1.59, 0.120 | -2.95, 0.005 | -1.16, 0.255 |
| Sophia |  |  |  |  | **5.33,**  **0.000** | **3.84, 0.000** | -0.78, 0.441 | 2.73, 0.009 |
| Diegosan |  |  |  |  |  | 0.47, 0.643 | **-6.36, 0.000** | **-5.15, 0.000** |
| Saya |  |  |  |  |  |  | **-4.73, 0.000** | **-3.58, 0.001** |
| Einstein |  |  |  |  |  |  |  | 3.58, 0.001 |


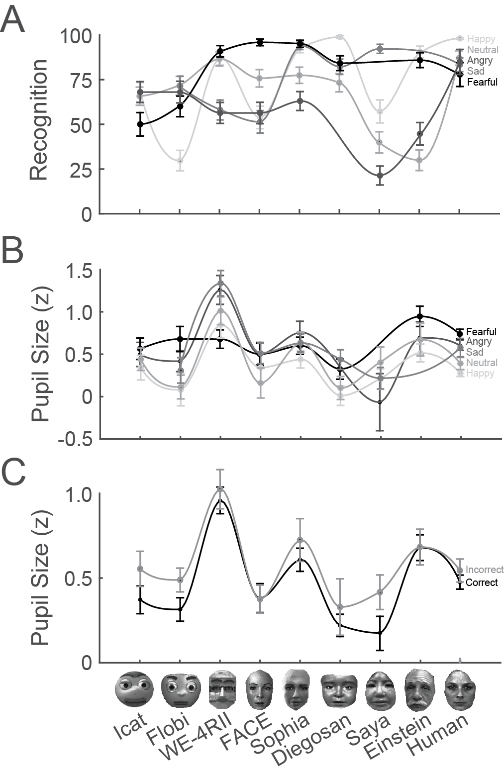


**Figure S2.** (A) Emotion recognition performance and (B) average pupil size between 1-3 seconds after stimulus onset per character as a function of human likeness (x-axis) and per emotional expression (grey value of lines). (C) Average pupil size per correctly (black) and incorrectly (grey) recognized emotions.

**Emotion and average pupil size analysis**

*Table S7. Results of ANOVAs per factor with agent and emotion as independent variables and pupil size as dependent variable*

| **Dependent variable (measure)** | **Independent variable (main effect*)*** | ***F*-statistic** | ***df*** | ***p*-value** |
| --- | --- | --- | --- | --- |
| Pupil Size | Agent | 0.439 | (1,39) | 0.512 |
|  | Emotion | 20.728 | (4,39) | <0.001 |
|  | Agent * Emotion | 2.396 | (4,156) | 0.053 |

*Table S8. Statistical comparisons of pupil size between human and robot faces*

| **Compared Emotions (pupil size: M±SD)** | | **Difference in pupil size (M±SD)** | ***t*-value** | ***p*-value (df = 39)** |
| --- | --- | --- | --- | --- |
| Fearful (0.65±0.34) | Angry (0.60±0.32) | 0.05±0.28 | 1.178 | 0.246 |
|  | Sad (0.53±0.36) | 0.11±0.29 | 2.516 | 0.016 |
|  | Neutral (0.41±0.35) | 0.24±0.30 | 4.911 | <0.001 |
|  | Happy (0.33±0.35) | 0.32±0.33 | 6.200 | <0.001 |
| Angry | Sad | 0.06±0.35 | 1.121 | 0.269 |
|  | Neutral | 0.18±0.23 | 5.046 | <0.001 |
|  | Happy | 0.27±0.27 | 6.214 | <0.001 |
| Sad | Neutral | 0.12±0.35 | 2.196 | 0.034 |
|  | Happy | 0.21±0.30 | 4.326 | <0.001 |
| Neutral | Happy | 0.08±0.30 | 1.759 | 0.086 |

**Emotion recognition analysis**

Average performance, measured as the percentage of correctly classified emotion expressions, was well above chance (M = 77.97%, SD = 4.35%, chance = 20%; *t*(39) = 84.28, *p* < 0.001), indicating that the task was not difficult to perform. A significant main-effect of emotion and agent (robot versus human) indicated that recognition performance differed across emotions and between humans and robot agents (Table S6). A significant interaction between emotion and agent further suggested that the pattern of recognition performance across emotions differed between human and robot faces. Participants recognized human emotional expressions better than the robots’ expressions (*t*(39) = 15.91, *p* < 0.001). Specifically, performance for all emotional expressions were significantly higher for human faces except for fearful expressions (Table S7). Within the human faces set, an happy expression was recognized best while fearful worst.

*Table S9. Results of ANOVAs for emotion recognition scores with agent and emotion as independent variables*

| **Dependent variable (measure)** | **Independent variable (main effect*)*** | ***F*-statistic** | ***df*** | ***p*-value** |
| --- | --- | --- | --- | --- |
| Emotion recognition performance | Agent | 11.977 | (1,39) | <0.001 |
|  | Emotion | 244.490 | (4,39) | <0.001 |
|  | Agent * Emotion | 27.950 | (4,156) | <0.001 |

*Table S10. Statistical comparisons of emotion recognition scores between human and robot faces*

| **Emotion** | **Mean±Sd**  **Human** | **Mean±Sd**  **Robot** | **Difference** | ***t*-statistic (df = 20)** | ***p*-value** |
| --- | --- | --- | --- | --- | --- |
| Sadness | 83.65±12.35 | 75.14±11.18 | 8.51±11.98 | 3.03 | 0.004 |
| Anger | 85.21±10.33 | 54.29±18.39 | 30.92±17.70 | 10.24 | <0.001 |
| Fear | 76.96±12.30 | 79.41±11.18 | -2.43±8.15 | 1.12 | 0.268 |
| Happiness | 98.33±3.24 | 72.60±13.93 | 25.73±13.47 | 12.08 | <0.001 |
| Neutral | 86.35±14.06 | 64.27±14.71 | 22.08±14.65 | 8.78 | <0.001 |

**
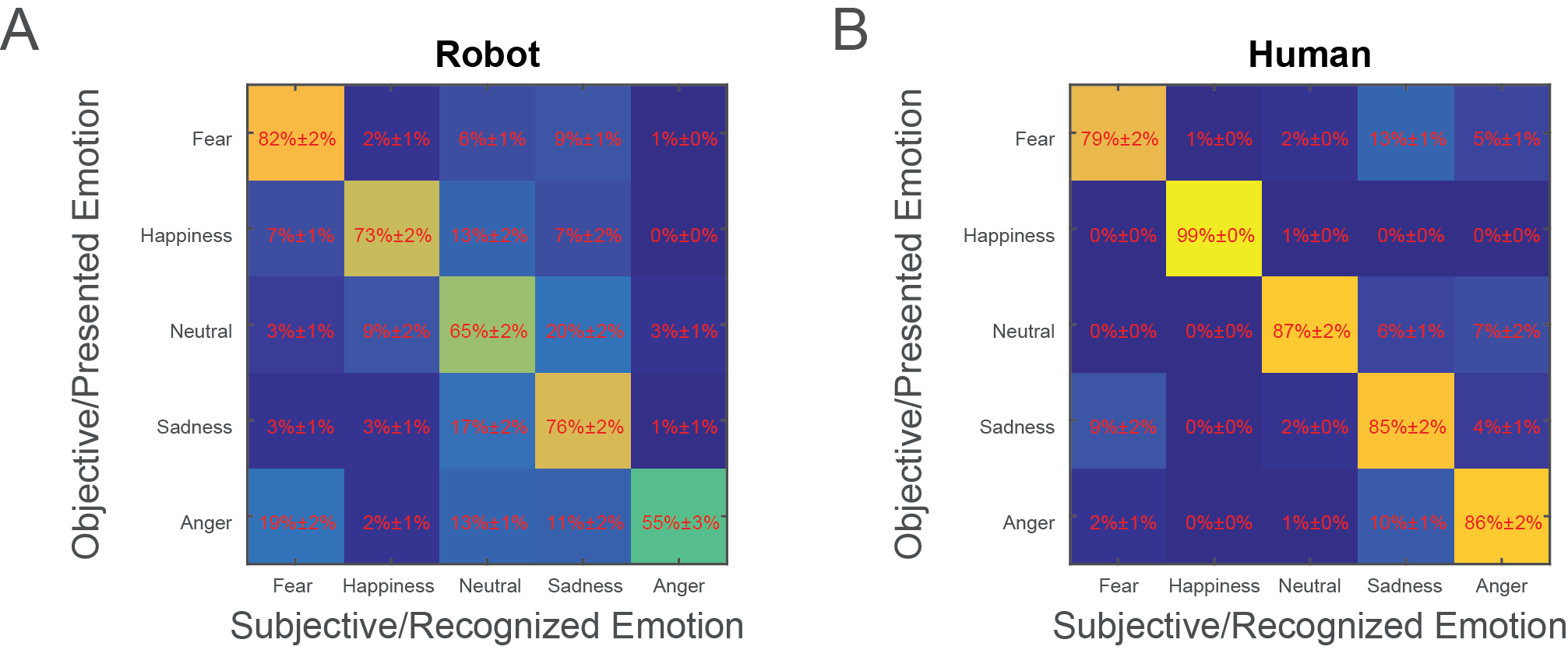
**

**Figure S3.** Confusion matrices for robotic (A) and human (B) emotions averaged across all characters. Each cell contains the average and standard error of the percentage correctly recognized emotion across participants per emotional expression. Color coding indicates frequency with yellow high frequencies and blue/green low frequencies.


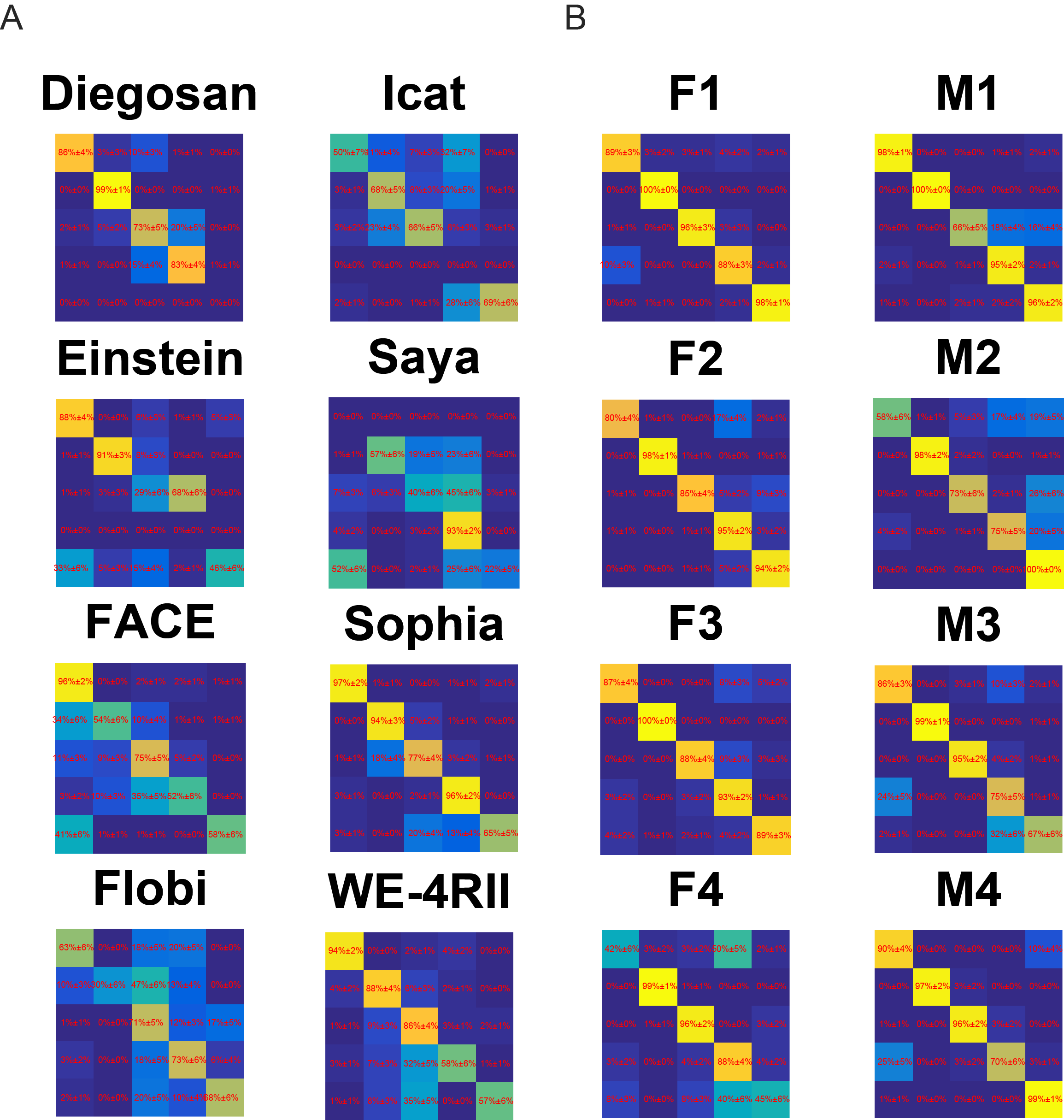


**Figure S4.** Same as Figure S3 but now confusion matrices for robotic (A) and human (B) emotions per character.

**Pupil pattern correlation analysis**


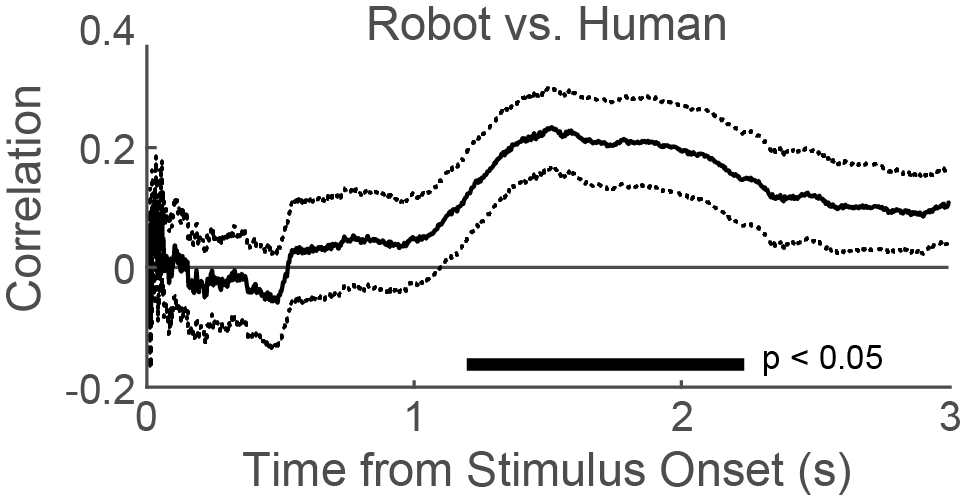


**Figure S5.** Correlation of average pupil size across emotions between human and robot stimuli. Significant correlations are indicated by the line at the bottom of the plot.
